# Algorithmic inclusion? The role of AI-assisted inclusive leadership in shaping gendered outcomes in multicultural academic teams

**DOI:** 10.3389/fpsyg.2026.1808574

**Published:** 2026-06-09

**Authors:** Iram Tahir, M. Sebnem Ensari, Khuram Shahzad, Rahat G. Kazmi, Ahmad Abu Arja

**Affiliations:** 1 University of Greater Manchester, Bolton, United Kingdom; 2 University of Lancashire, Preston, United Kingdom; 3 Global Banking School, London, United Kingdom

**Keywords:** AI-assisted inclusive leadership, algorithmic bias, gender equality, Higher Education Institutions, multicultural teams, transformative governance

## Abstract

Artificial Intelligence (AI) adoption across Higher Education Institutions (HEIs) has gained considerable momentum, and is now being used for faculty recruitment, promotion, workload allocation, research impact evaluation and student assessments. Notwithstanding the objectivity and efficiency that AI brings, its deployment within historically gendered academic institutions raises concerns about the perpetuation and reproduction of structural inequalities. This conceptual paper proposes a governance framework for AI-assisted inclusive leadership focused on gender equality in multicultural teams. AI systems operate within institutional contexts and mediate the relationship between leadership, governance and equity. The paper proposes four institutional solutions; human-in-the-loop deliberation, transparent AI protocols, routine equity audits, and interdisciplinary oversight committees, as mechanisms for mitigating algorithmic bias and enhancing institutional reflexivity. The paper also introduces a four-stage governance maturity framework as an iterative approach to ethical AI inclusion in inclusive leadership of multi-cultural teams. The paper argues that only through a strategic thought-leadership approach to AI inclusion in Higher Education Institutions can structural gender inequalities in multicultural teams be addressed through redesigning of productivity metrics, recognition of emotional and invisible labour, and by addressing gender-embeddedness in institutional systems.

## 1 Introduction

Inclusive leadership remains a critical and central issue when studying multicultural teams, as the effective working of multicultural teams depends significantly on openness, accessibility and psychological safety, all tenets of inclusive leadership (Carmeli et al., 2010; Khan et al., 2020; Nishii, 2013; Randel et al., 2018). This is particularly relevant in academic contexts where teaching, research, and governance is increasingly characterised by teams that have overlapping national, cultural, and disciplinary boundaries, and therefore, inclusive leadership becomes essential for ensuring equitable participation, high team performance, and inclusive acknowledgement. In the current scenario, where Higher Education Institutions (HEIs) are increasingly moving towards global transformation and the use of AI-assisted technologies in work, the role of inclusive leadership becomes even more central to ensuring equitable access to people from different backgrounds. AI-assisted technologies are increasingly being used for activities such as meeting transcription systems, automated performance dashboards, workload modelling systems, grant writing assistance, predictive funding analytics, and student evaluation analysis platforms.

Technologically, AI-assisted work is innovative in the way that it enhances transparency, coordination across teams, and the quality of decision making. However, at the same time, the fact that HEIs are structurally gendered institutions (Acker, 1998), the question of gender equity remains central to the use of AI-assisted technologies. Evidence already exists that points to gender disparities in research funding allocation, leadership representation, authorship visibility, promotion rates, and service burden distribution (Benschop and Brouns, 2003; European Commission, 2021; Guarino and Borden, 2017). These inequalities are embedded in institutional logics, evaluation systems, and definitions of merit, rather than solely in individual bias. At the same time, research suggests that AI systems have been trained on historical data that frequently encode and amplify structural inequalities (Birhane, 2021). This means that while AI algorithmic systems may appear objective, they could invariably reproduce power asymmetries because of these being embedded in training datasets and optimization criteria.

As a result, two sets of literature have emerged in parallel on the topic. Whereas research on inclusive leadership suggests it is an essential determinant of relational inclusion in multicultural teams, scholarship on AI governance research focuses on the need to mitigate bias and ensure fairness in sociotechnical systems (Barocas et al., 2023; Leslie et al., 2024). However, there is still a literature gap on the integration of these two different perspectives to examine how inclusive leadership helps the deployment and management of AI-assisted technologies within gendered institutions, such as the Higher Education sector.

This research article addresses this gap by asking the following question:

### 1.1 In what circumstances does AI-assisted inclusive leadership in multicultural teams in Higher Education Institutions reduce or reproduce gendered inequalities?

This is a conceptual study, and it argues that in the presence of AI-assisted technology use, inclusive leadership no longer remains a relational competence issue but rather assumes responsibility for sociotechnical stewardship. This means that leaders, in the interest of pursuing inclusivity, must go beyond fostering psychological safety and belongingness, and also ensure that algorithmic infrastructures are fair in shaping evaluation, visibility, and opportunity. In this paper, ‘AI-assisted work’ refers to the use of computational systems used to supplement, guide and/or partially automate decision making, coordination and evaluation processes in the Higher Education settings. These include rule-based systems (for, e.g., work allocation), Machine Learning systems (used for predictive performance dashboards), and Generative AI systems (used for grant-writing, for e.g.). In all three contexts, there is a risk of bias reproduction and blurred boundaries, particularly in machine learning and generative AI, as these rely on historical data patterns and probabilistic outcomes. AI-assisted inclusive leadership refers to leadership practices that integrate the above-mentioned tools in team governance whilst maintaining openness, fairness, transparency and accessibility in team participation and evaluation. Here, this concept emphasises the human governance of AI systems for sustained inclusion. AI literacy, later propositioned, refers to an individual’s ability to understand, interpret, and critically assess AI outcomes, and includes technical elements as well as an awareness of issues such as bias, responsible use and limitations.

This paper proposes a dual-path model that suggests how AI in multicultural academic teams can either act as an innovation that amplifies and helps inclusion or is manifested as a structural mechanism that reproduces and reinforces gender inequality. The model conceptualises AI not only as a main theoretical construct, but also as a contextual innovation that redefines inclusive leadership practice and suggests new issues that inclusive leadership needs to address in an AI assisted workplace.

This research paper integrates inclusive leadership theory and gendered organisation theory with the concepts of multicultural team and algorithmic governance to develop an understanding of the innovation, challenges, and solutions for inclusive leadership in AI-assisted multicultural, gendered contexts.

## 2 Methodology

### 2.1 Research design

This study follows a conceptual theory-building approach methodologically, examining the influence of AI-assisted inclusive leadership on gendered inclusion and performance in multicultural teams in the Higher Education sector. A conceptual approach was suitable for this topic as it is an emerging domain, and empirical evidence is fragmented, with multiple studies lacking meaningful integration (Jaakkola, 2020).

### 2.2 Theoretical integration

We develop a model through the integration of four literature bodies, including: (1) inclusive leadership theory incorporating psychological safety, belongingness and voice (Carmeli et al., 2010; Nishii, 2013); (2) gendered organisation theory, highlighting the structural inequalities in institutions (Acker, 1990); (3) algorithmic and AI fairness research, examining bias and decision-making authority in AI systems (Barocas et al., 2023; Selbst et al., 2019); and (4) multicultural teams, emphasizing cultural moderators such as power distance and gender egalitarianism (House et al., 2004; Stahl et al., 2010).

### 2.3 Model development

The model was developed in three stages: First, key constructs across the four literature bodies were identified; second, core mechanisms, such as psychological safety, algorithmic contestation and institutional gender embeddedness were specified; and third, the Dual-Path Sociotechnical Model was constructed, identifying distinct pathways for amplification and reproduction. The propositions have been presented to facilitate empirical testing with future studies.

### 2.4 Scope and analytical approach

The focus is on Higher Education Institutions, multicultural teams and AI-assisted contexts. A sociotechnical perspective is adopted, highlighting the interaction between leadership practices, institutional structures and AI technologies (Kalluri, 2020).

The model presented here is not empirically tested but is intended to be a guide for future empirical studies.

### 2.5 Literature identification and synthesis

Rather than employing a conventional systematic literature review methodology, this study adopts a theory-building and integrative conceptual approach, as advocated by Jaakkola (2020). The key sources of literature were accessed from academic databases including Scopus, Google Scholar, Web of Science and EBSCO Business Source Complete. Time period covered was from 1990 to 2025 to reflect both seminal organisational context works and the more recent emerging works in AI.

The key search terms used included combinations and variations, including synonyms, for ‘inclusive leadership’, ‘multicultural teams’, gender inequality’, ‘higher education’, ‘gendered organisations’, ‘AI governance’, ‘algorithmic bias’, ‘algorithmic fairness’ and ‘sociotechnical stewardship’.

Inclusion criteria prioritised studies addressing leadership, inequality, gender, and AI inclusion and governance. Key seminal works, research within organisational contexts, and more recent peer-reviewed works were given preference, especially for AI focus. Exclusion criteria included studies focusing purely on the technical aspects of AI, AI studies without organisational contexts embedded within them, and context-specific studies that lacked generalisable insights.

The four core literatures—inclusive leadership, gendered organisation theory, AI governance, multicultural teams—were used due to the complementary nature of these works in addressing relational, cultural, structural and technological dimensions of inclusion and equality. An iterative synthesis of this literature led to the development of the Dual Path Model presented in this paper.

## 3 The gendered nature of Higher Education Institutions

### 3.1 Gendered organisation theory and academic institutions

Acker (1998) maintains that gendered assumptions are embedded in organisational structures, norms, and practices with some sectors exemplifying these more than others, such as academic institutions. In the Higher Education sector, there are five key mechanisms through which gendered organisational dynamics are characterised. Firstly, there is the gender division of labour, where women academics engage disproportionately more in mentoring, advising, and committee services, and this usually comes at the expense of research productivity (Guarino and Borden, 2017). This then, secondly, impacts performance metrics and evaluation systems, which prioritise publication counts and grant income, and women lose out because of the low value attributed to relational labour (Williams et al., 2013). Thirdly, symbolic leadership norms are usually aligned with masculine-coded traits such as assertiveness, competitiveness, and uninterrupted career trajectories (Eagly and Karau, 2002; Heilman, 2012), which are quite different to the lived experiences of women (academics). Fourthly, Rudman and Glick (2001) point towards interactional bias and backlash, where women academics face double standards and penalties for norm violations much more significantly than their male counterparts. Finally, Mitchell and Martin (2018) suggest that student evaluations contain systematic gender bias affecting career advancement. These patterns demonstrate that inequality is structurally embedded, rather than episodic. The study adopts an inclusive understanding of gender, recognising the experiences of women, non-binary, trans, and other gender-diverse academics. Also, the understanding of the intersection between gender, class, race, disability and migration status is considered, allowing for a more accessible opportunity in multicultural academic environments.

### 3.2 Multicultural contexts and cultural moderators

Gendered institutional logics intersect with cultural norms when it comes to managing multicultural academic teams. House et al. (2004) used the GLOBE framework to highlight cross-cultural variation in power distance, collectivism, uncertainty avoidance, and gender egalitarianism. This could have multiple interpretations. In high power-distance contexts, algorithmic authority may supersede other mechanisms of establishing inclusivity. In low gender-egalitarian contexts, women’s leadership may be contested more; and in more collectivist contexts, the contestation of even outputs may be discouraged to uphold relational harmony. At this point, inclusive leadership provides the buffer that is needed to mitigate these effects by providing psychological safety to members of multicultural teams (Stahl et al., 2010). Simultaneously, however, AI introduces standardized evaluation logics, which may be contradictory to the local norms and institutional culture. This multiculturality intensifies the need for structural, gender-aware regimens in AI-assisted governance and leadership of teams.

## 4 AI as a structurally embedded institutional actor

Although discussions of AI and its purported uses within HEIs is a seemingly growing area of discussion and intrigue (Bagshaw, 2025: Gillani et al., 2023: Khairullah et al., 2025: Parwana, 2025: QAA, n.d.), caution should be exercised prior to its wholesale adoption. Whilst the past several years has seen HEIs grapple with the implications of AI within, beyond, and between universities, the student and staff bodies, and wider society, its integration demands careful consideration across multiple areas, variously spanning the social, political, ethical, and cultural. Instead of permitting a particular discourse of AI to become normative, it is important that we do not regard AI within HEIs as a neutral tool. Rather, AI should be regarded as a structurally embedded social actor: a system that operates to (re)shape conceptions of—and relationships with—authority, evaluation, and legitimacy. As HEIs look to adopt these (socio)technical systems, the means by and through which authority, evaluation, and legitimacy are (re)constituted will become simultaneously more complex and, resultantly, opaque. This poses problems for the way(s) in which current gendered inequalities are (un)accounted for and subsequently addressed.

These problems arise through algorithmic authority and deference; algorithmic ‘fairness’ and structural abstraction, and; trust, power, and sociotechnical systems. Responses to AI fall on a continuum, with aversion and deference on the two ends, and appreciation in between. Aversion refers to resistance following observed errors, whereas deference occurs when users accept AI outputs without critically questioning them, assuming their authority; appreciation denotes trust for data-driven tasks performed by AI. These responses are governed by task complexity, perceived objectivity, and cultural contexts. In multicultural teams, high power-distance may encourage deference, whereas low power distance may foster contestation and aversion.

### 4.1 Algorithmic authority and deference

The adoption and application of AI technologies within organizations, including HEIs, poses questions of and for leadership teams as various tasks are either wholly or partially handed over to AI systems and the black box processes these involve (Quaquebeke and Gerpott, 2023). Dietvorst et al. (2015) points to the fact that there is a propensity for individuals to defer to algorithmic recommendations even when they are flawed. The focus on data-driven and objective decision-making might give epistemic authority to AI outputs without their consideration in a more cultural and social context. In multicultural teams, cultural power-distance norms further shape this deference (House et al., 2004). Sycophantic systems also develop an environment of over-agreement, power-worshipping, and optimization for approval, rather than truth or justice (Sharma et al., 2023). They also avoid challenging authority, which could be problematic in the management of multicultural teams, particularly from an inclusive leadership point of view. This issue of algorithmic fairness also supersedes social contexts by reproducing inequality in the interest of predictive accuracy (Fazelpour and Lipton, 2020).

### 4.2 Trust, power, and sociotechnical systems

Lee and See (2004) suggest that when people have a high level of trust in automated systems, they accept its outputs without questioning it, and start relying on it instead of contesting or factchecking it. In the context of inclusive leadership, over-trust on AI systems would mean that an AI tool would evaluate performance and screen candidates for leadership potential, rather than the tacit knowledge and sociocultural idiosyncrasies vested in human cognitive abilities. This could potentially lead to strengthening gender and cultural bias and structural inequalities because of the high trust in AI outputs and low contestation of these, allowing bias to get institutionalised. Kalluri (2020) and Benjamin (2023) provide a sociotechnical perspective to this, and under this perspective, AI is no longer a neutral efficiency tool; instead, it characterises what valuable knowledge is, what institutional values hold more merit, and potentially amplifies some voices over others.

## 5 Inclusive leadership as sociotechnical stewardship

Inclusive leadership, defined by leaders’ openness, accessibility, and availability (Carmeli et al., 2010), fosters psychological safety and creates an environment where people feel a sense of belonging and uniqueness (Brewer, 1991; Nishii, 2013; Randel et al., 2018), encouraging individuals to speak up and participate in work processes. As such, voice and participation become central outcomes (Smyth, 2009; Qi and Liu, 2017; Li and Hang, 2017). Mir et al. (2021) also link inclusive leadership to innovative work behaviour and project success.

This understanding gains further depth when examined through Sociotechnical Systems Theory and Foundational Feminist Technology Theory.

Sociotechnical Systems Theory, by Trist and Bamforth (1951), states that organisational outcomes result from the interaction between technical systems and social structures. Technologies are inseparable from human practices and institutional power, with their impact determined by interpretation, implementation, and governance. Therefore, technology’s support for inclusion depends more on leadership practices than on technological features alone.

Foundational Feminist Technology Theory (Wajcman, 1991; Faulkner, 2001; Suchman, 2007) also challenges the assumption of technological neutrality. Specifically, it argues that technological systems are shaped by gendered power relations, historical inequalities, and dominant institutional norms. Building on this perspective, research demonstrates that technologies often reproduce masculine ideals of efficiency, productivity, and control. Yet, they also have the potential to promote inclusive leadership when designed and governed in reflexive, ethical, and context-sensitive ways. In this view, technology is not just a tool for automating leadership processes; rather, it constitutes a domain in which power relations are either reinforced or transformed.

Building on these theoretical insights, this study extends existing frameworks by introducing the concept of “sociotechnical stewardship” as a central dimension of inclusive leadership in AI-mediated multicultural teams. Sociotechnical stewardship, for the purpose of this paper, is defined as the capacity of leaders to control and manage the relationship between social and technological systems in ways that enhance inclusion and equity, across the dimensions of transparency, contestability, equity auditing, and reflexive governance. Sociotechnical stewardship emphasises leaders’ responsibility not only for managing interpersonal relations but also for the ethical, critical, and context-sensitive governance of AI and algorithmic systems within organisational decision-making. This approach reconceptualises inclusive leadership as a sociotechnical practice, shifting the focus from individual leader intentions to the active shaping, interpretation, and mobilisation of technological systems to achieve inclusion, equity, and fair performance outcomes.

### 5.1 Psychological safety and algorithmic contestation

Edmondson (1999) defines psychological safety as a shared belief that a team environment supports interpersonal risk-taking. Within this framework, psychological safety refers to the collective perception among team members that they can voice concerns, challenge assumptions, and express doubts without fear of negative consequences. In AI-supported decision environments, where artificial intelligence systems assist or influence team decisions, complexity and uncertainty increase due to the opacity, authority, and perceived objectivity of algorithmic outputs. Psychological safety allows team members to critically evaluate AI-generated recommendations, facilitating rigorous assessment of the accuracy, fairness, and contextual relevance of AI outputs rather than accepting them as inherently correct.

AI-mediated decision environments introduce additional complexity through algorithmic authority, opacity, and perceived objectivity. These factors may inhibit critical engagement with AI-generated outputs.

In this context, psychological safety becomes essential for enabling team members to question the accuracy, fairness, and contextual appropriateness of algorithmic recommendations without fear of negative interpersonal consequences. Previous research demonstrates that inclusive leadership behaviours, including openness, accessibility, and the active encouragement of diverse perspectives, are primary antecedents of psychological safety (Nembhard and Edmondson, 2006; Carmeli et al., 2010). By signalling that questioning is legitimate and valued, inclusive leaders reduce the interpersonal risks associated with challenging dominant viewpoints, including those presented by algorithms.

At the same time, research on employee voice indicates that individuals are least likely to speak up when outcomes are perceived as objective or beyond challenge, a condition characteristic of algorithmic decision-making (Morrison, 2011). Experimental evidence further shows that individuals often defer to algorithmic advice unless they feel sufficiently safe to challenge it (Dietvorst et al., 2015), while algorithmic appreciation reinforces the tendency to accept AI outputs as unquestionable truths (Logg et al., 2019). The opacity of machine learning systems further deepens this reluctance to question algorithmic decisions (Burell, 2016).

Taken together, these findings suggest that inclusive leadership fosters psychological safety, which, in turn, enables the critical evaluation of AI outputs by legitimising dissenting voices and reducing the perceived risks of contesting algorithmic decisions.

Cultural contexts further shapes the extent to which psychological safety translates into voice and the willingness to challenge algorithmic outputs. In high power-distance cultures, hierarchical norms discourage questioning authority, making individuals less likely to challenge not only leaders but also algorithmic systems that are perceived as authoritative decision-makers (Hofstede, 2001; Botero and Van Dyne, 2009). As a result, even when inclusive leadership fosters psychological safety, the expression of dissent may remain constrained.

In contrast, cultures characterized by high gender egalitarianism emphasize equality, participation, and the legitimacy of diverse perspectives, thereby encouraging open communication and voice behaviors (House et al., 2004; Eagly and Wood, 2012). In such contexts, psychological safety is more likely to translate into active questioning and critical evaluation of AI-generated recommendations.

Finally, in high uncertainty avoidance cultures, individuals tend to prefer structured and predictable solutions and are more likely to rely on formalized systems to reduce ambiguity (Hofstede, 2001; Taras et al., 2010). In AI-mediated environments, this tendency may manifest as greater reliance on algorithmic outputs, thereby reducing the likelihood of contesting AI recommendations even when psychological safety is present (Xu and Gao, 2025; Jarrahi, 2018).

Accordingly, we propose the following inclusive leadership mechanisms:

P1: Inclusive leadership has a direct and moderating positive relationship with psychological safety of multicultural teams in an AI-mediated environments, with it being weaker in high power-distance cultures, stronger in high gender-egalitarian cultures, and weaker in high uncertainty avoidance cultures.

P2: Psychological safety in multicultural teams mediates the relationship between inclusive leadership and algorithmic contestation, with it being weakened in high power distance contexts, strengthened in high gender egalitarian contexts, and weakened in high uncertainty avoidance.

### 5.2 Belongingness, uniqueness, and digital visibility

Randel et al. (2018) demonstrate that inclusive leadership behaviours directly enhance employees’ experiences of belongingness and uniqueness. However, these processes occur within technology-mediated evaluation contexts. Systems of quantification fundamentally reshape what is visible and valued in organisations, as numerical indicators often displace contextual, relational, and less easily measurable forms of contribution (Espeland and Stevens, 2008; Muller, 2018). In AI-mediated work settings, this effect intensifies, with algorithmic performance metrics that may reduce contributions to standardised outputs alone. Leonardi and Treem (2020) further argue that contemporary digital and AI-enabled systems considerably increase behavioral visibility by making the actions of individuals, teams, and technologies observable to third parties through quantified, aggregated, and algorithmically ordered data. They emphasise that visibility is not a neutral reflection of work but a sociometrical performance formed by aggregate quantification, algorithmic ordering, and observers’ interpretive inference. This process produces paradoxical consequences: while visibility may enhance coordination and accountability, it can also narrow definitions of valuable work and obscure context, effort, and relational labour. Building on these understandings, this analysis proposes that AI systems reshape visibility in ways that risk reducing contribution to numerical outputs unless leaders actively intervene. Accordingly, inclusive leaders’ reflexive, critical, and contextual interpretation of AI-generated performance measures is essential to counteracting reductionism and sustaining inclusion.

The interpretation of such AI-generated visibility is shaped by cultural dimensions. In high power-distance cultures, algorithmic outputs are more likely to be accepted as authoritative, limiting critical reinterpretation. In high uncertainty avoidance cultures, reliance on structured and predictable systems increases dependence on algorithmic metrics to reduce ambiguity. In contrast, low power-distance and low uncertainty avoidance contexts are more conducive to questioning and reinterpreting these outputs. Accordingly, inclusive leaders play a critical role in reflexively contextualizing AI-generated metrics to preserve belongingness and uniqueness, with the effectiveness of this process contingent on cultural values that shape reliance on, and contestation of, algorithmic authority.

Accordingly, we propose P3 and P4:

P3: Belongingness and uniqueness among gender diverse members in multicultural teams can be enhanced through a reflexive and socio-culturally appropriate interpretation of AI-generated metrics by inclusive leadership in a moderated mediation effect, with it being strengthened when inclusive leaders contextualise AI outputs, stronger in high gender-egalitarian contexts, and stronger in high uncertainty avoidance contexts.

P4: The relationship between inclusive leadership and equitable AI outcomes is moderated by algorithmic literacy, with higher literacy strengthening bias detection and reducing unquestionable reliance and acceptance of AI outcomes. This will be stronger in low power-distance cultures, and weaker in high uncertainty avoidance contexts.

## 6 The dual-path sociotechnical model

The cultural contexts are important in the interpretation of AI systems, and this model integrates cultural dimensions as integral moderators that shape AI-assisted leadership dynamics. We use the GLOBE framework (House et al., 2004) to identify power distance, gender egalitarianism and uncertainty avoidance as the key moderators that would influence algorithmic deference and contestation. For, e.g., high power-distance would reduce the potential of contesting AI outcomes, whereas high gender egalitarian contexts would increase bias sensitivity in AI systems. These cultural dimensions would influence AI contribution to amplification or reproduction pathways.

Based on the preceding discussion, we propose two possible pathways:

### 6.1 Amplification path (innovation)

This pathway uses AI as an equity accelerator, and AI governance is used to enhance and ensure inclusion through leadership interventions. This would be fostered by the following conditions:

1. *Strong Inclusive Leadership* would call upon leaders to solicit and support diverse perspectives and enable shared expectation and decision-making. Leaders would value the relational and emotional labour that women typically put more effort towards, and would also be the change agents and change champions in challenging biased systems. This aligns with inclusive leadership as this cannot be symbolic, and helps realign voice and authority with the changing dynamics.
2. *High Psychological Safety* would provide the environment in institutions where people would feel comfortable in questioning AI outputs, raise their concerns about apparent bias, admit uncertainty where relevant, and challenge leadership decisions if needed. This aligns with inclusive leadership values of openness, accessibility and psychological safety. When people do not have this psychological safety, AI outputs may be accepted without challenge, which provides AI with unquestionable authority and undermines human instinct and leadership.
3. With *Transparent Governance*, AI systems can be built with inherent audits, have explanatory value, and are open to revision through appeals and feedback. This would also reduce power asymmetry, which is essential to the effectiveness of multicultural teams, and central to inclusive leadership.
4. *Equity Audits* will move from being symbolic reviews to measures of vigilance, allowing for a monitoring of gendered performance ratings, promotion rates, work allocation models, and any scoring disparities that might have been generated, or reinforced, with AI algorithms.
5. *Institutional Reflexivity* will compel institutions to question embedded assumptions in metrics, question beneficiaries of current productivity definitions, and recognise forms of invisible labour. This would enable for a more appropriate encoding of AI algorithms to ensure that AI dos not encode what institutions already value.

The implementation of these measures is expected to lead to the following outcomes:

- *Recognition of invisible labour* would enable the identification of emotional support work, measure collaboration contributions, and quantify coordination efforts, all of which are, historically and typically, feminised labour, allowing it to become visible and rewardable.
- *Equitable workload redistribution* will help identify people who have an overloaded service work allocation, who perform diversity labour, and who carry relational burdens within organisations, allowing AI to help re-balance this instead of silent exploitation.
- *Reduced bias in evaluation* will allow AI-enabled auditing and reflexivity to ensure that gendered language and reviews are flagged, promotion criteria is standardised, and any algorithmic disparities are corrected to ensure equity.

These cultural dimensions not only shape whether AI governance mechanisms are enacted, but also how they are interpreted and legitimized within organisational contexts. In low power-distance settings, governance practices such as audits, appeals, and transparency are more likely to be seen as participatory safeguards, encouraging distributed responsibility for identifying and correcting bias. In contrast, in high power-distance cultures, such mechanisms may be perceived as extensions of authority rather than tools for contestation, thereby limiting their use as instruments of equity. Similarly, in high uncertainty avoidance contexts, formal governance structures may increase trust in AI systems by signalling control and reliability, yet paradoxically discourage ongoing questioning.

Based on the above discussion, the following proposition is presented:

*P5:* High inclusive leadership combined with transparent AI governance would lead to greater gender equity.

The key mechanism here would be the use of inclusive leadership to moderate AI interpretation and use. Transparent governance would prevent algorithmic authority over equity norms, leading to an inclusive culture which is amplified by AI assistance, not restricted by it. This would manifest more in high gender-egalitarian contexts, and less in high uncertainty-avoidance and high power-distance contexts, where existing norms are reinforced.

### 6.2 Reproduction path (structural challenge)

In this pathway, AI becomes the inequality stabiliser, where it does not necessarily create bias, but does institutionalise existing bias, under the following conditions:

1. *Weak inclusive leadership* would mean that leaders would not challenge existing norms. They would treat outputs generated by AI as a neutral truth without human input, and would prioritise efficiency over fairness. It would also put off difficult equity conversations within teams, which would not only have an impact on the inclusive nature of leadership in multicultural teams, but also reinforce structural bias, though passively.
2. *High institutional gender embeddedness,* derived from institutional theory, would reinforce the norms of the ‘ideal worker’ (the always available, hyper-productive archetype), the masculine coded leadership archetypes, the undervaluation of emotional labour and penalization of caregiving, all of which hinder women’s progress and development. AI algorithms that have been developed within these environments will reproduce these mechanisms.
3. *Low governance transparency* would manifest in the form of black box models, lack of audit publications, lack of explanation access, and lack of formal structured appeals processes, which entrenches power in gender inequality norms.
4. *High algorithmic deference* pushes people to assume that because it is an algorithm, it must be right; which means that managers defer to risk ratings, performance scores, and productivity dashboards. While in and of themselves these issues are not problems, the lack of human discretion means that vested power remains where it is, and this would lead to perpetuating inequality.

These measures are projected to lead to the following outcomes:

- *Reinforced biased metrics* would continue to measure productivity with logged hours or output quantity, penalising care responsibility, undervaluing collaboration, and punishing interruptions. AI assistance in these situations would optimise the advantage that certain groups already have over others, without providing voice to the marginalised ones.
- *Entrenchment of productivity norms* would increase hypervisibility because of AI monitoring, leading to increased performance and conformance pressures, rewarding presenteeism, and penalising flexibility, all of which are ideal worker norms, which would further be enforced by AI.
- *Separation of voice* would be experienced because of high algorithmic authority and low psychological safety, ultimately leading to fewer complaints, reduced dissent, self-blame, and silent tolerance of inequity, leading to a system that is fragmented and broken, but might give a false sense of propriety. This is an antithesis to multicultural teams and inclusive leadership.

These dynamics are further conditioned by cultural values that shape how algorithmic authority and institutional norms are enacted and contested. In high power-distance cultures, both managerial decisions and AI-generated outputs are more likely to be accepted without challenge, reinforcing the institutionalisation of existing gender hierarchies through algorithmic systems. Similarly, in high uncertainty avoidance contexts, strong preferences for predictability and formalisation increase reliance on algorithmic decision-making as a source of perceived objectivity, further reducing the likelihood of questioning biased metrics or governance structures. In contrast, cultures characterised by high gender egalitarianism are more likely to problematise entrenched norms and question biased outputs, thereby interrupting the passive reproduction of inequality. As a result, the extent to which AI stabilises or amplifies existing inequalities depends not only on leadership and governance conditions but also on cultural norms that either reinforce or challenge the legitimacy of algorithmic authority.

Building on the above discussion, the following proposition is formulated:

*P6*: Weak inclusive leadership in a high institutional gender embedded environment would intensify inequality in an AI governance environment.

The key mechanism here would be the operationalization of existing gendered structures with AI. It would mean that there would be no reinterpretation or intervention from leadership, allowing algorithmic governance to amplify embedded inequality and institutional bias. This will manifest more in high power-distance and high uncertainty-avoidance cultures where challenging AI decisions is less likely to happen, and less in high gender-egalitarian contexts where bias is more likely to be questioned and challenged.

For the amplification pathway, a minimum threshold of transparency, algorithmic literacy, and psychological safety would be required, whereas systems that have low transparency, strong structural inequality, and low contestability will lead to the reproduction pathway.

**Figure 1:**
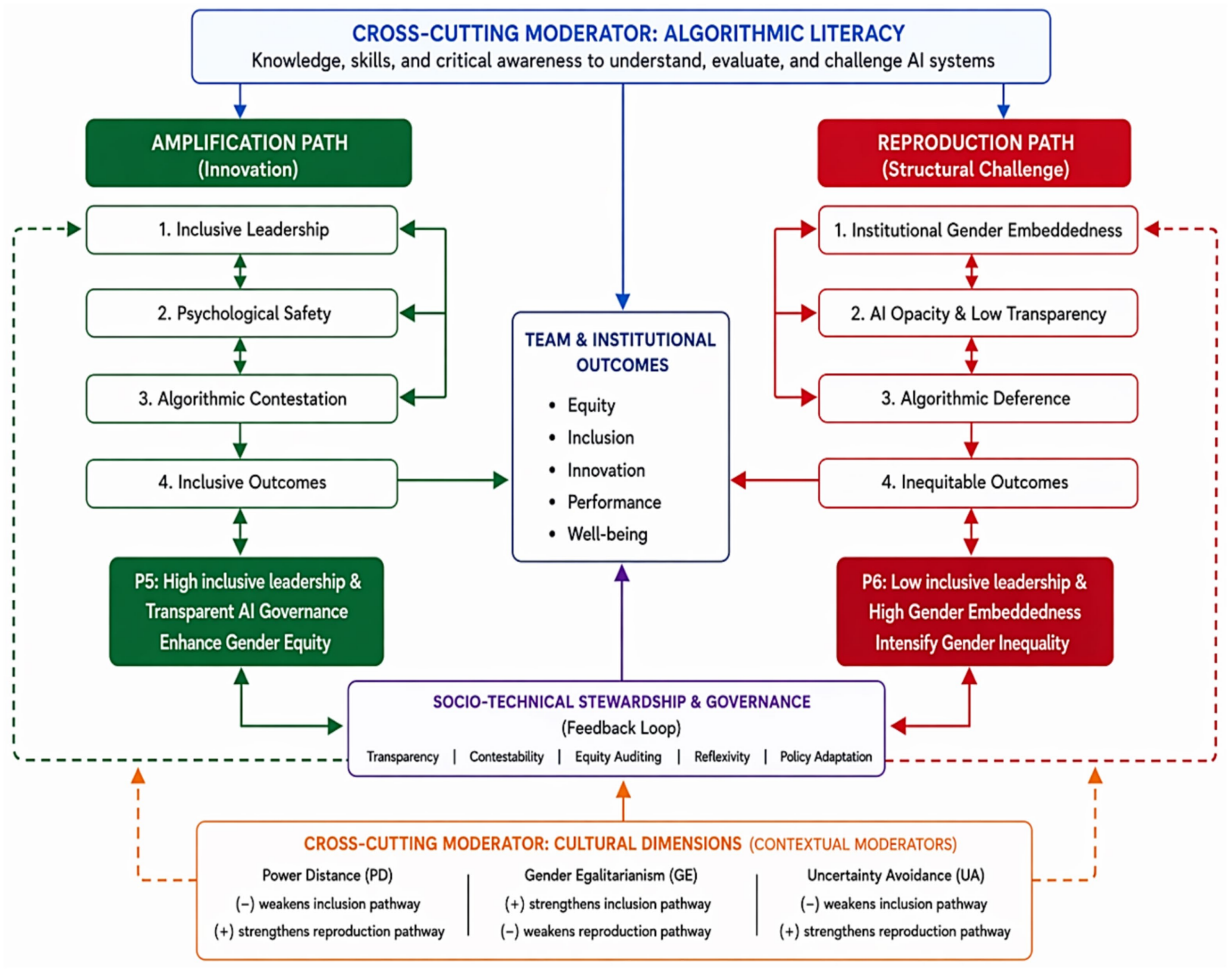
The dual path sociotechnical model. Source: self-created by the authors.

As a result, the dual-path sociotechnical model is illustrated in Figure 1.

The conditions within each pathway are conceptualised as interacting factors. Inclusive leadership, psychological safety, and algorithmic awareness and contestation, combine to produce inclusive/amplification outcomes through reinforced feedback loops. Similarly, institutional gender embeddedness, algorithmic opacity/low transparency, and algorithmic deference interact to produce inequitable/reproduction outcomes. For both pathways, algorithmic literacy and cultural dimensions function as influencing moderators.

## 7 Governance solutions for inclusive AI leadership

Based on the work presented in this paper, we argue that AI-assisted inclusive leadership in the Higher Education sector requires deliberate governance structures that mediate the interaction between algorithmic systems and institutional gender dynamics. We propose the following as institutional solutions for this, and we posit that the combined efforts that come from these mechanisms will provide the foundation of a governance maturity framework that would then determine whether AI will be used as a reproductive or transformative factor in relation to gender equality.

1. *Human-in-the-Loop Deliberation:* AI is increasingly used in HEIs to rank job applicants, score research impact, and analyse teaching evaluations. By keeping humans in the loop, faculty committees would be able to review AI outputs with a human lens and would be able to interrogate algorithmic metrics rather than accepting them automatically. This would allow for contextual factors, such as service burdens and care responsibilities, to be considered. Female faculty take on disproportionate service burdens and mentoring roles, experience productivity gaps due to care responsibilities, and receive gender-biased teaching evaluations—human deliberation would allow for adjustment to these mediators and correct the evaluation bias to be gender neutral. AI would be in an informative rather than determinative role. When AI is embedded within human deliberations, institutions can check and correct the mechanistic reinforcement of gendered productivity norms.
2. *Transparent AI protocols* would allow HEIs to clearly articulate the operationalization of AI systems, including data sources, calculation of performance metrics, weightage of research impact scores, testing of gender bias, and the appeals process. Transparency is critical in academic institutions, upholding legitimacy and fairness being core values. This would also help question masculine-coded norms of ideal academic productivity, and reduce structural invisibility, helping gender equality.
3. *Routine equity audits* using parity metrics will enable gender-focused monitoring to evaluate gender disparities in AI-driven hiring recommendations, teaching evaluation score gaps, workload allocation patterns, and ground allocation models. These recurring audits will help measure false negative rates in AI screening that are gender relevant, bias in NLP of recommendation letters, as well as evaluation of gendered language patterns in student evaluations. This would enable institutions to proactively identify and address disparities.
4. *Interdisciplinary oversight committees* will represent faculty from gender studies, data scientists, legal scholars, HR, union and student representatives, and diversity officers, who will allow the existing expertise to be institutionalised in governance. This would provide a sociological insight in gender equality, would create awareness for legal compliance, would allow for a feminist analysis of institutional norms and behaviours, and would allow for an input of lived experiences of female members of faculty to be incorporated.

The governance maturity would determine pathway outcomes. Reflexive governance would increase the likelihood of amplification pathway, while low governance maturity would reinforce the reproduction pathway. Feedback loops would enable progression towards more inclusive systems, with measures such as audits and appeals processes allowing staff to challenge decisions playing a critical role.

However, governance mechanisms designed to promote inclusion might have some unintended detriments. For example, routine audits may be seen as additional and unnecessary surveillance, and in some cases, might incentivize performative compliance rather than a sincere commitment. Similarly, these requirements may create additional administrative work that might affect an already overburdened workforce, and particularly service staff may be disproportionately affected, which is already disproportionately represented by female faculty members. Similarly, appeals processes may favour individuals with greater institutional knowledge, or those that have a greater confidence in challenging decisions.

To mitigate these risks, it is essential that institutions ensure governance mechanisms are proportionate, participatory, and aligned with the workflow distribution. This could take on the form of rotating responsibilities for oversight, simplified reporting processes, and a reflexive evaluation of the governance structures.

## 8 Proposed governance maturity framework

**Figure 2:**
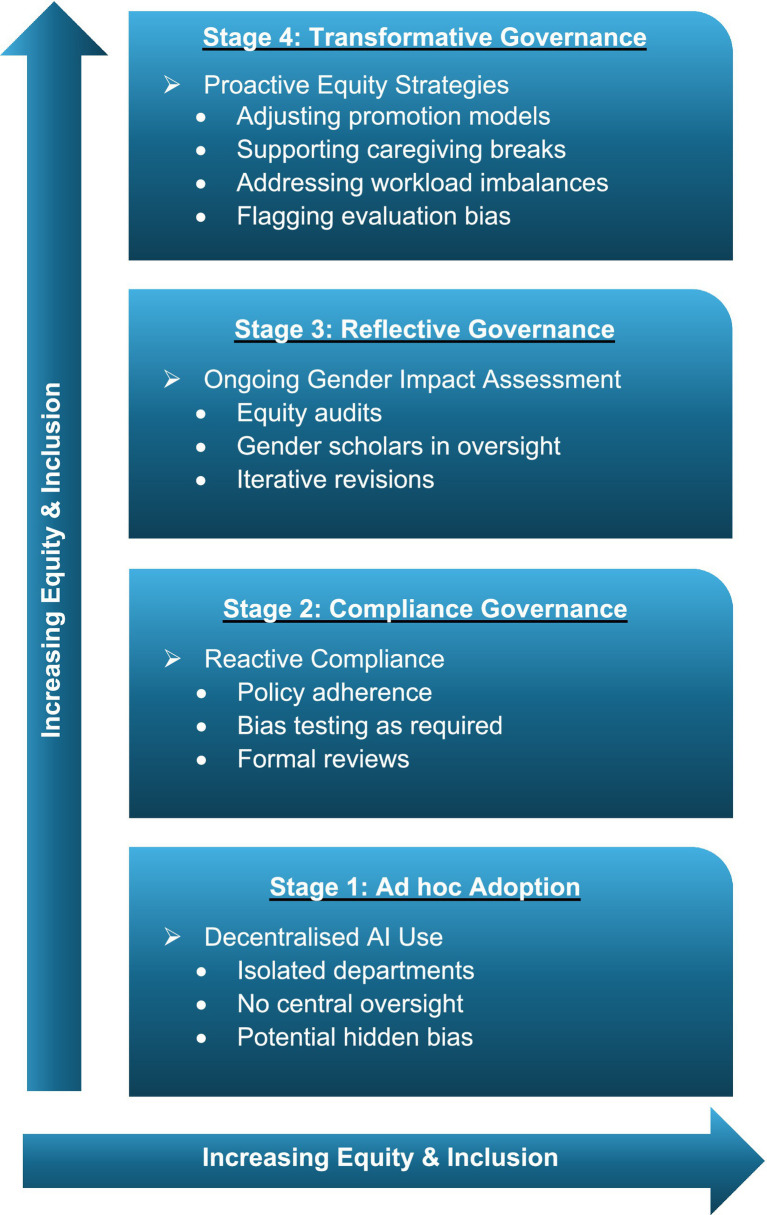
Proposed governance maturity framework. Source: self-created by the authors.

The governance framework, as illustrated in Figure 2, progresses through 4 stages of maturity as exhibited below:

*Stage 1: Ad-Hoc Adoption:* Characterised by initiation, is unmanaged and reactionary—At this stage, there would be an *ad hoc* adoption of AI tools. This would be characterised by departments independently adopting AI tools without a central policy. Decentralisation remains key. This stage risks hidden gender bias because of decentralised governance and disciplinary silos.

*Stage 2: Compliance Governance*: Characterised by foundational level work—At this stage, policies will be developed to comply with anti-discrimination laws, and bias will be tested only when legally required. It will have a formal review process, but gender equality would be more of a reactive initiative, rather than a proactive one.

*Stage 3: Reflective Governance:* Characterised by proactivity and proficiency, this stage would see ongoing gender impact assessment becoming the norm, with the inclusion of gender scholars in oversight committees. Faculty consultation processes would also be encouraged to allow for several iterative revisions of AI systems being used in the institution. This kind of reflexive governance would allow institutions to self-examine their own systems and correct them as necessary.

*Stage 4: Transformative Governance:* Characterised by strategic foresight, thought leadership and institutionalised action—transformative governance would use AI intentionally to correct structural inequalities, which could mean adjustment of promotion models, allowing for caregiving interruptions, performance algorithms being weighted for mentoring and service burdens, identification of gendered workload imbalances, flagging of bias in evaluations, and providing research support for underrepresented faculty. At this stage, AI moves beyond a measure of fairness, to a measure of corrective action.

The dual-path sociotechnical model presented above correlates with these four stages. The *Ad Hoc* and Compliance stages align with the Reproduction path, and Reflexive and Transformative stages align with Amplification and Innovation Path.

## 9 Empirical operationalisation

Inclusive leadership can be measured using validated scales (Carmeli et al., 2010). Psychological safety can be measured using Edmonson’s (1999) scale, and algorithmic literacy may be assessed through knowledge-based or self-report measures. In this context, behavioral indicators would include the frequency of AI output challenges (psychological safety), the representation of parity metrics (equity outcomes) and governance transparency indicators. Multi-level modelling can be used to test interactions between leadership, institutional contexts, and AI systems.

## 10 Theoretical contributions

This article makes several important theoretical contributions. Firstly, it extends leadership theory in sociotechnical contexts, with AI being a mediator. It also integrates gendered organisation theory with algorithmic governance framework. It develops a Dual Path Sociotechnical model, and proposes a Governance Maturity Framework that would enable a fairer and more equitable AI use within HEIs. It also reframes inclusion and inclusive leadership as institutional design capacity, lending a human perspective to AI-assisted leadership.

## 11 Limitations

A key limitation of this study is its conceptual nature, and it does not provide empirical validation. It focuses on the Higher Education context, limiting generalizability. While intersectionality is acknowledged, it is not fully modelled. Much of the literature is based and studied in Western contexts.

Future reach research could empirically test the model across different cultural contexts and also examine longitudinal AI adoption effects. There is also the opportunity to conduct a further intersectional analysis in depth.

## 12 Conclusion

AI-assisted inclusive leadership in multicultural academic teams presents an opportunity for a shift in practice, allowing inclusive leaders to act as sociotechnical stewards within structurally gendered institutions. Given that AI does not inherently democratise institutions and may perpetuate bias, inclusive leadership needs to evolve towards algorithmic flexibility and institutional design capacity. This would allow AI to become a tool for equity, rather than a mechanism of inequality.
